# Regulation of Phagocytosis in Macrophages by Membrane Ethanolamine Plasmalogens

**DOI:** 10.3389/fimmu.2018.01723

**Published:** 2018-07-24

**Authors:** Julio M. Rubio, Alma M. Astudillo, Javier Casas, María A. Balboa, Jesús Balsinde

**Affiliations:** ^1^Instituto de Biología y Genética Molecular, Consejo Superior de Investigaciones Científicas (CSIC), Universidad de Valladolid, Valladolid, Spain; ^2^Centro de Investigación Biomédica en Red de Diabetes y Enfermedades Metabólicas Asociadas (CIBERDEM), Madrid, Spain; ^3^Departamento de Bioquímica y Fisiología, Universidad de Valladolid, Valladolid, Spain

**Keywords:** ethanolamine plasmalogen, phagocytosis, arachidonic acid, inflammation, monocytes/macrophages

## Abstract

Macrophages, as professional phagocytes of the immune system, possess the ability to detect and clear invading pathogens and apoptotic cells through phagocytosis. Phagocytosis involves membrane reorganization and remodeling events on the cell surface, which play an essential role in innate immunity and tissue homeostasis and the control of inflammation. In this work, we report that cells deficient in membrane ethanolamine plasmalogen demonstrate a reduced capacity to phagocytize opsonized zymosan particles. Amelioration of plasmalogen deficiency in these cells by incubation with lysoplasmalogen results in a significant augmentation of the phagocytic capacity of the cells. In parallel with these increases, restoration of plasmalogen levels in the cells also increases the number and size of lipid rafts in the membrane, reduces membrane fluidity down to levels found in cells containing normal plasmalogen levels, and improves receptor-mediated signaling. Collectively, these results suggest that membrane plasmalogen level determines characteristics of the plasma membrane such as fluidity and the formation of microdomains that are necessary for efficient signal transduction leading to optimal phagocytosis by macrophages.

## Introduction

Macrophages play key functions in innate and inflammation by clearing pathogens, damaged cells, and debris *via* highly regulated phagocytosis processes, which aim at efficiently restoring tissue homeostasis (1, 2). To carry out this function, macrophages are endowed with a variety of cell surface receptors, collectively called opsonin-independent receptors, which include the C-type lectins such as dectin-1 and the mannose receptor; scavenger receptors such as SR-A-I, SR-A-II, MARCO, and CD36; and toll-like receptors such as TLR-2 and TLR-6 (3–6). To improve recognition of phagocytosable material, pathogens, damaged cells, or debris can be opsonized by immunoglobulins and complement and be recognized by opsonin-dependent receptors such as the Fc receptors FcγRI, FcγRII, and FcγRIII, and the complement receptors CR1, CR3, and CR4 (6–8). Engagement of phagocytic receptors, both non-opsonic and opsonic, triggers the synthesis and release of an ample variety of mediators such as cytokines, chemokines, and arachidonic acid-derived eicosanoids that regulate the inflammatory response (3–8).

A number of phagocytic receptors have been localized or are recruited upon activation to membrane microdomains called lipid rafts, including both non-opsonic (TLR-2, TLR-6, and dectin-1) and opsonic (FcγR and CR3) (9–16). Lipid rafts are highly dynamic and tightly ordered membrane microdomains enriched in cholesterol, glycosphingolipids, and glycosylphosphatidylinositol-linked proteins. Lipid rafts are involved in numerous cell functions, which include cell signaling, membrane sorting and trafficking, migration, cell adhesion (17–19), as well as phagocytic and pathogen entry processes (20, 21).

Plasmalogens are also ubiquitously found within lipid rafts (22). These are glycerophospholipids with a vinyl ether bond in the *sn-1* position of the glycerol backbone. Thus the *sn-1* position in plasmalogens is occupied by a fatty alcohol, not a fatty acid as it is common for most membrane glycerophospholipids. Ethanolamine is the most frequent headgroup present in mammalian plasmalogens (23). Macrophages are rich sources of ethanolamine plasmalogens, which localize primarily in the inner leaflet of the plasma membrane and present an unusual enrichment in polyunsaturated fatty acids, most notably arachidonic acid, in the *sn-2* position (23–25). Given the importance of macrophages as major sources of arachidonate-derived eicosanoids as mediators of inflammation, the key role of ethanolamine plasmalogens in arachidonic acid mobilization reactions has been long recognized (23, 26, 27). Because of the vinyl ether bond of ethanolamine plasmalogens, these phospholipids have also been found to play roles as endogenous antioxidants and in the regulation of plasma membrane biophysical properties such as fluidity, fusion tendency, and thickness (28, 29).

Previous work from our laboratory has investigated the mechanisms regulating phospholipid turnover *via* deacylation/reacylation reactions during phagocytosis, as well as the phospholipase A_2_ forms involved (30–41). Results from these studies have identified discrete lipid metabolites whose synthesis appears to be associated with specific stimulation conditions and thus, may allow the identification of specific attributes of the immune response as regards the lipid pathways and metabolites involved (30–41). In this study, we have applied similar mass-spectrometry-based lipidomic and confocal microscopy approaches to investigate the role of plasmalogens during phagocytosis. Taking advantage as well of the use of plasmalogen-deficient cells (42, 43), we show that reduction in plasmalogen levels leads to altered phagocytosis of opsonized zymosan (OpZ) particles by macrophages which can be attributed to changes in the plasma membrane fluidity and the formation and functioning of the lipid rafts. We further show that these alterations can be significantly reversed when cellular plasmalogen levels are increased by incubating the cells with lysoplasmalogens, which incorporate into the cells and restore the cellular plasmalogen pool.

## Materials and Methods

### Reagents

Dulbecco’s modified Eagle’s medium (DMEM) was from Lonza (Basel, Switzerland). Zymosan A, labeled with Alexa Fluor 594, and cholera toxin B subunit (recombinant) labeled with Alexa Fluor 647 were from Molecular Probes (Carlsbad, CA, USA). Chloroform and methanol, 2-propanol, *n*-hexane and ammonium acetate (HPLC grade) were from Fisher Scientific (Hampton, NH, USA). Lipids and standards for mass spectrometry were purchased from Avanti Polar Lipids (Alabaster, AL, USA), Cayman (Ann Arbor, MI, USA), or Larodan Fine Chemicals (Malmoe, Sweden). The antibodies against phosphorylated and non-phosphorylated extracellular signal-regulated kinases p44/p42 were from Cell Signaling (Amherst, MA, USA). All other reagents were from Sigma-Aldrich.

### Cell Culture Conditions

RAW264.7 macrophage-like cells and the ether phospholipid-deficient RAW.108 cells (a generous gift from Dr. R.A. Zoeller, Boston University) (42, 43) were grown in DMEM supplemented with 10% (v/v) fetal bovine serum, 100 U/ml penicillin, 100 µg/ml streptomycin, and 2 mM l-glutamine at 37°C in a humidified atmosphere of 5% CO_2_ at 37°C, exactly as described (31, 37). For experiments, the cells were treated in DMEM without serum for 1 h before addition of the various stimulants for the indicated concentrations and periods of time. Zymosan particles were prepared as described elsewhere (44, 45). Only zymosan preparations that were shown not to contain endogenous phospholipase A_2_ activity, as measured by enzyme assay (46–50), were used in this study. Cell protein was measured according to Bradford (51), using the BioRad kit.

### Phagocytosis Assay

Opsonized zymosan, labeled with the fluorophore Alexa Fluor 594, or opsonized latex beads (3 µm particle size) were used as a stimulus. The zymosan particles and latex beads were opsonized by incubation with murine serum for 20 min at 37°C, at a ratio of 1 ml serum per 10 mg particles (38). The cells were seeded over glass coverslips, allowed to adhere, washed with DMEM, and resuspended in this medium. OpZ or opsonized latex beads were added and, after a 30-min incubation at 37°C, coverslips were washed with PBS and transferred to plates at 37°C, and phagocytosis was allowed to proceed for 30 min. Reactions were stopped by fixation with 4% paraformaldehyde in PBS containing 3% sucrose for 15 min. Afterward, paraformaldehyde was removed by washing the cells three times with PBS. DAPI staining was carried out by treating cells with this dye at a concentration of 1 µg/ml in PBS for 10 min. Coverslips were mounted on microscopy slides with 10 µl a polyvinyl alcohol solution until analysis by fluorescence microscopy. A Leica TCS SP5 X confocal microscope with white laser (470–670 nm) (Leica Microsystem, Wetzlar, Germany) was used for these studies. Images were analyzed with LAS AF v. 2.6.3 (Leica) and ImageJ (National Institutes of Health, USA; http://rsb.info.nih.gov/ij/). The phagocytic index was calculated by dividing the number of phagosomes by the total number of cells in a field, which was multiplied by the percentage of phagocytosing cells, as described elsewhere (52).

### Flow Cytometry Analyses

The cells were incubated with latex beads (yellow-green fluorescent FluoSpheres^®^ Microspheres of 1 µm size, 488 nm spectral line; 5–7 particles/cell) (Molecular Probes™) or OpZ (labeled with Alexa Fluor 488; 5–7 particles/cell) for 30 min at 37°C. Afterward the cells were washed thrice with PBS to remove the particles, and fresh medium was added to allow phagocytosis to complete. Then the cells were washed again with PBS and harvested with TrypLE™ Express Enzyme (Gibco, Thermo Fisher). Cell fluorescence was quantified by flow cytometry using a Beckman Coulter Gallios cytofluorometer. Data were analyzed with the Kaluza software, version 1.5a.

For the analysis of apoptotic cell phagocytosis by macrophages, apoptotic Jurkat T cells were used. Apoptotic cell death was induced in these cells by treating them with 500 µM H_2_O_2_ for 20 h (53, 54). Afterward, the Jurkat cells were stained with 50 µM propidium iodide for 20 min. After washing the cells twice with PBS, target cells were added to the RAW 264.7 or RAW.108 macrophage monolayers in a final volume of 1.5 ml of DMEM. The macrophages had previously been stained with CellTracker™ Deep Red Dye (ThermoFisher). The plates were centrifuged at 300 *g* for 3 min so as to bring the target cells into direct contact with the macrophages. The ratio of target cells to macrophages was 3:1. The phagocytosis reaction was allowed to proceed for 2 h in a humidified CO_2_ incubator at 37°C, after which the macrophages were harvested with TrypLE™ Express Enzyme. The macrophages were then washed twice with PBS and analyzed by flow cytometry using a Beckman Coulter Gallios cytofluorometer. Deep red fluorescence from CellTracker™ Deep Red Dye was analyzed in FL6, while red fluorescence from propidium iodide was analyzed in FL3. To quantify phagocytosis, red fluorescence was analyzed only in the cell populations exhibiting significant deep red fluorescence (i.e., the macrophages). Macrophages are double positive for red and deep red fluorescence only if they ingested the propidium iodide-labeled Jurkat cells.

### Analysis of Lipid Rafts

When needed, RAW cells were stained for lipid raft visualization with Alexa Fluor 647-labeled cholera toxin B subunit (CT-B) following the manufacturer’s experimental protocol (Molecular Probes, Carlsbad, CA, USA). Cells were washed twice with cold PBS and fixed in 4% paraformaldehyde in PBS containing 3% sucrose for 15 min. After several washes, the glasses were placed and mounted onto slide for microscopy.

For all microscopy measurements, the staining pattern of cells from three separate and independent experiments was considered (20–35 cells total per treatment). The size of lipid rafts was determined by analyzing particles that were ≥0.1 μm^2^ with ImageJ (55). This threshold was selected because an area smaller than 0.1 µm^2^ results in domains that could not be accurately measured. The lipid raft number was determined for each cell and averaged for all cells for a given treatment. The data were then averaged for all cells per treatment group from all the independent measurements.

### Fluorescence Recovery After Photobleaching (FRAP)

The cells were maintained in imaging medium (HBSS pH 7.4, 25 mM HEPES, 1.3 mM CaCl_2_, and 1.3 mM MgCl_2_) at 37°C with 5% CO_2_ in a cage incubator attached to the microscope. Images were captured using an oil immersion, 63×, 1.4 NA, HCX PL APO CS objective in a confocal microscope at 37°C (TCS SP5X; Leica). Briefly, a rectangular region of interest of 6 µm^2^ (3 × 2 µm) was defined within the plasma membrane for effective bleaching, 650 nm white laser was set at 100% power for a pulse of 0.834 s. 10 Prebleach images and 100 postbleach images were acquired at 20% of the maximum laser power, with a 256 × 256 pixels resolution, 8 bits and full scanning speed at 1,000 Hz every 0.278 s. Pinhole size was optimized to 238.8 µm aperture. A minimum of 40 cells per condition was analyzed with ImageJ software. FRAP measurements were full-scale normalized according to the method described by Giakoumakis et al. (56). The resulting data were fit to a single exponential curve model, and the mobile fraction of plasma membrane was calculated (56–58).

### Lipid Analysis by Mass Spectrometry

Analysis of phospholipids by liquid chromatography coupled to mass spectrometry was carried out as described elsewhere (34–41), using an Agilent 1260 Infinity high-performance liquid chromatograph equipped with an Agilent G1311C quaternary pump and an Agilent G1329B Autosampler, coupled to an API2000 triple quadrupole mass spectrometer (Applied Biosystems). Phospholipid molecular species were identified by comparison with previously published data (34–41). Lipid analysis by gas chromatography coupled to mass spectrometry was carried out exactly as described elsewhere (59–63), using an Agilent 7890A gas chromatograph coupled to an Agilent 5975C mass selective detector operated in electron impact mode, equipped with an Agilent 7693 Autosampler and an Agilent DB23 column (60 m length × 0.25 mm internal diameter × 0.15 µm film thickness).

### Immunoblot Analysis

This was carried out exactly as described elsewhere (63, 64). The immunoblots were visualized using enhanced luminescence. Densitometry was performed on scanned images using Quantity One^®^ software (Bio-Rad), and values were normalized for the corresponding controls of each experiment.

### Statistical Analysis

All experiments were carried out at least three times with incubations in duplicate or triplicate, and the data are expressed as mean ± SE. Statistical analysis was carried out by Student’s *t*-test, with *p* < 0.05 taken as statistically significant.

## Results

**Figure 1:**
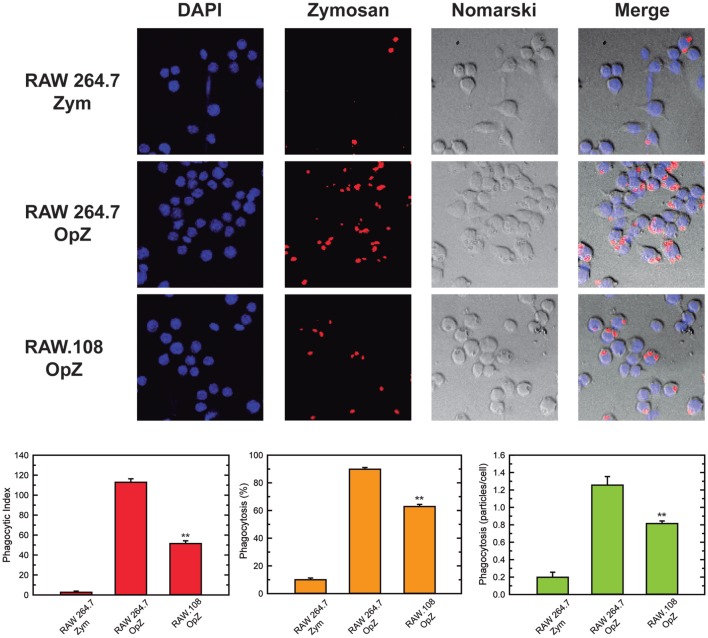
Phagocytosis of zymosan particles in RAW 264.7 and plasmalogen-deficient RAW.108 cells. The cells were exposed to fluorescent non-opsonized (Zym) or opsonized zymosan (OpZ) particles for 30 min, as indicated (10 particles per cell, equivalent to approximately 0.2 mg per million cells), and analyzed for phagocytosis of fluorescent zymosan particles by confocal microscopy (red color, middle columns). DAPI (1 µg/ml) was used to mark the nuclei (blue, left columns). Nomarski and merge images are also shown (right columns). The average of three independent experiments with 20 determinations each is shown (mean ± SEM) (bottom panel). Original magnification, ×20. **Significantly different (*p* < 0.01) from RAW 264.7 cells exposed to OpZ.

**Figure 2:**
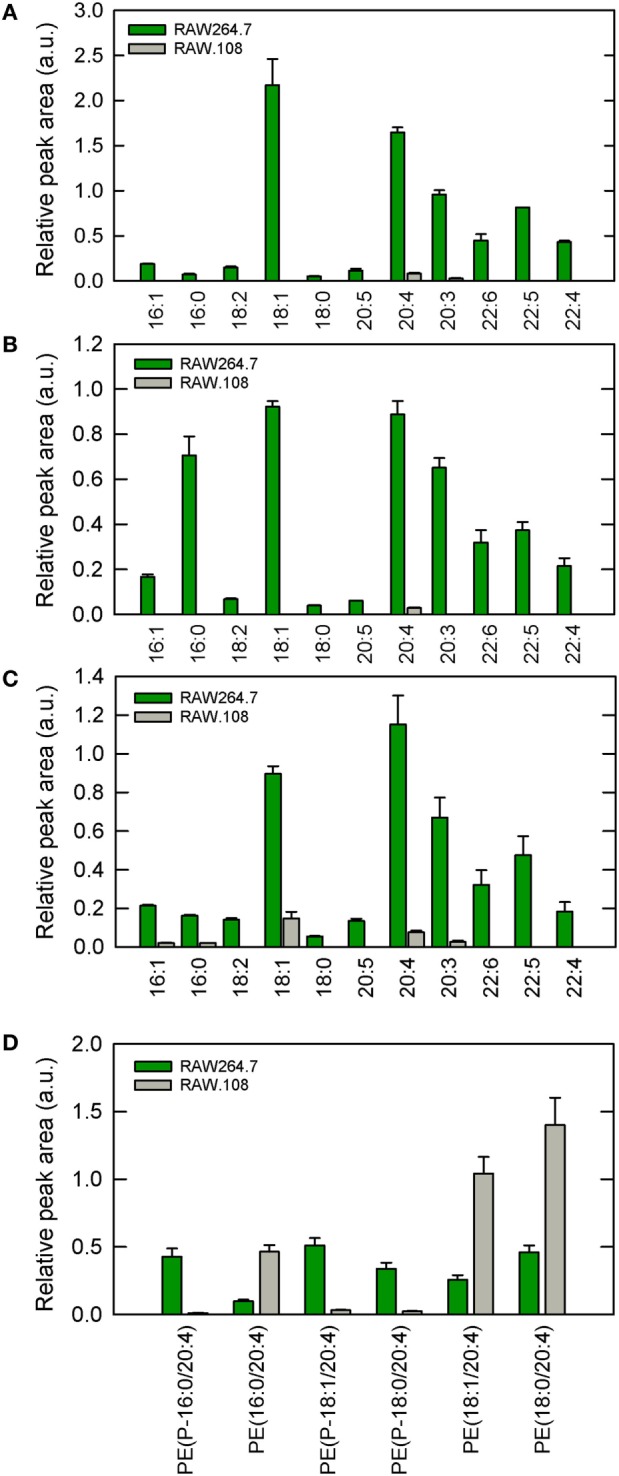
Ethanolamine plasmalogen content of RAW 264.7 and RAW.108 cells. Analyses were carried out by liquid chromatography coupled to mass spectrometry. The fatty acid distribution within plasmalogen species containing hexadecyl (P-16:0) **(A)**, octadecenyl (P-18:1) **(B)**, or octadecyl (P-18:0) **(C)** moieties at *sn-1* position is shown. Fatty acids (abscissa) are abbreviated as carbon number:double bonds. In **(D)**, the distribution of arachidonic acid (20:4) moieties in ethanolamine phospholipids (including plasmalogen and diacyl species) in RAW 264.7 cells versus RAW.108 cells is shown. Results are given as mean ± SEM of three independent experiments carried out in duplicate.

In previous work from our laboratory, we demonstrated that phospholipase A_2_-mediated hydrolysis of ethanolamine-containing phospholipids at the membrane is a key event to support phagocytosis of yeast-derived zymosan and live bacteria by human macrophages (40). To explore further the involvement of ethanolamine-containing membrane phospholipids in phagocytosis, in this work we took advantage of the use of an ethanolamine plasmalogen-defective variant of the RAW 264.7 cell line, called RAW.108 (42, 43). RAW 264.7 macrophage-like cells are extensively utilized as a prototypical model for studies on macrophage metabolism and signaling (65, 66). These cells phagocytize non-OpZ particles rather poorly, which is believed to be due to a very low expression level of dectin-1 in these cells (3). However, opsonization of the zymosan with serum, which allows recognition of the particles by complement receptors-1, -3, and -4, and Fcγ receptors (5–8), results in the cells phagocytizing the stimulus to a much greater extent (Figure 1). Interestingly, phagocytosis of OpZ particles was significantly impaired in the ethanolamine plasmalogen-deficient RAW.108 cells (Figure 1). Comparative lipidomic analyses of wild-type cells versus RAW.108 cells by mass spectrometry confirmed the practically complete absence of plasmalogens in the latter (Figure 2). Importantly, however, the total amount of cellular arachidonate-containing ethanolamine phospholipid was preserved, because of a compensatory elevation of non-plasmalogen ethanolamine phospholipids (i.e., the diacyl species) (Figure 2).

To determine whether the phagocytic response of RAW.108 cells could be restored by exogenously increasing cellular plasmalogen levels, we preincubated the cells with 10 µM lysoplasmenylethanolamine (1-*O*-1′-octadecyl-2-lyso-*sn*-glycero-3-phosphoethanolamine, lysoPlsEtn), so that the cells incorporated and reacylated it, thus replenishing their cellular plasmalogen pool. Under our assay conditions in cell culture media, the critical micellar concentration for the lysophospholipids used in this study was estimated to be between 15 and 20 µM. This result, which is similar to data by others (67), indicates that at the concentrations used the lysophospholipids are primarily in monomeric form. This avoids undesired nonspecific effects stemming from the formation of aggregates of varying sizes and shapes that could interact with the cells (40).

**Figure 3:**
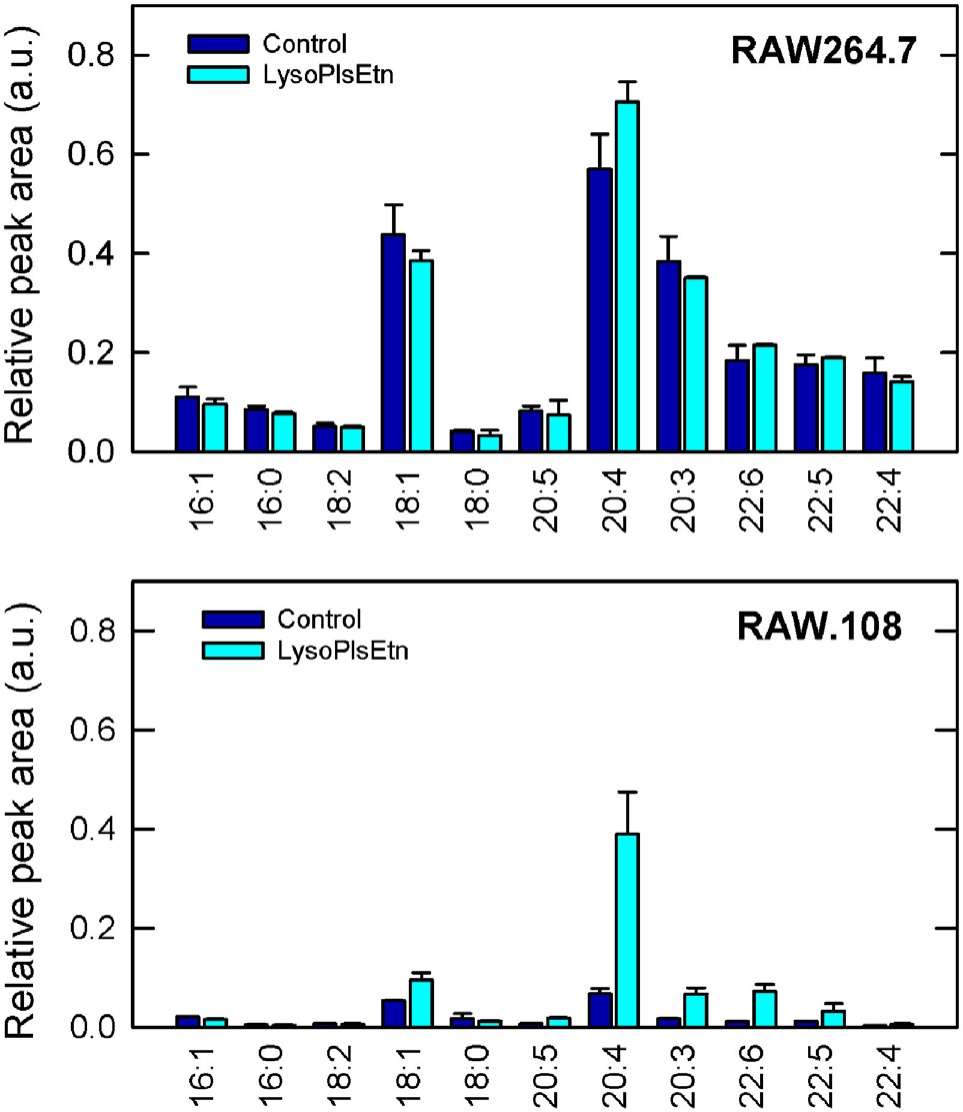
Incubation of RAW.108 cells with lysoPlsEtn increases plasmalogen content. RAW 264.7 cells (top panel) or RAW.108 cells (bottom panel) were incubated without (dark blue bars) or with 10 µM lysoPlsEtn (cyan bars) for 40 min. Afterward, cellular plasmalogen content was determined by liquid chromatography coupled to mass spectrometry. Results are given as mean ± SEM of three independent experiments carried out in duplicate.

Figure 3 shows that treatment with lysoPlsEtn significantly elevated the amount of plasmalogen in the RAW.108 cells, especially that containing arachidonic acid (20:4), which increased to levels close to 60–70% of those of normal RAW 264.7 cells. Interestingly, addition of lysoPlsEtn to normal RAW 264.7 cells increased only slightly the amount of cellular ethanolamine plasmalogen in these cells, suggesting that the capacity of this pool to expand is limited and, in turn, that normal RAW 264.7 cells already contain the requisite plasmalogen that is necessary for cell function (Figure 3).

**Figure 4:**
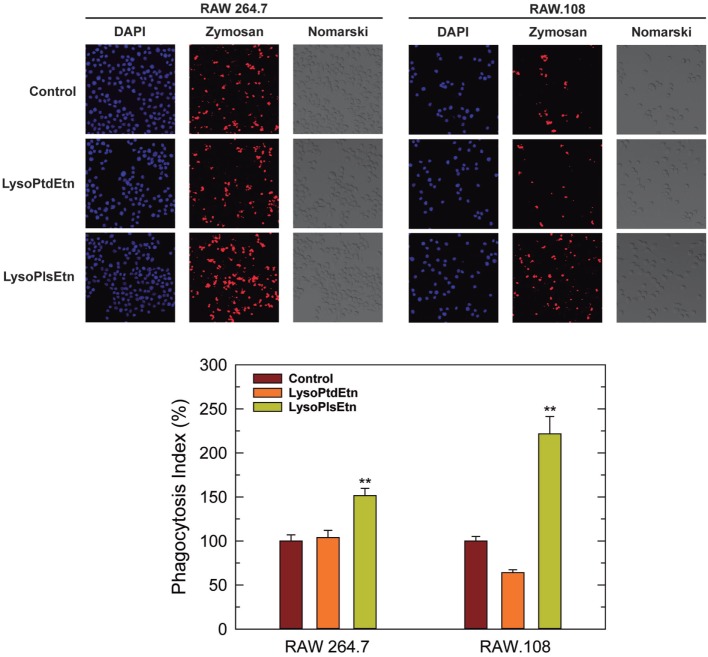
Effect of lysoPlsEtn on the phagocytosis of opsonized zymosan by RAW 264.7 and plasmalogen-deficient RAW.108 cells. The cells were either untreated (brown bars) or treated with 10 µM lysoPlsEtn (orange bars) or 10 µM lysoPtdEtn (yellow bars) for 10 min. Afterward, fluorescent zymosan particles (10 particles per cell) were added for 30 min, and phagocytosis was analyzed by confocal microscopy (red color, middle columns). DAPI (1 µg/ml) was used to mark the nuclei (blue; left columns). Nomarski images are also shown (right columns). The average of three independent experiments with 20 determinations each is shown in the bottom panel (mean ± SEM). Phagocytic indexes in the bottom panel are given as percent values with respect to controls without added lysophospholipids to allow for direct comparison between the responses of RAW 264.7 cells versus RAW.108 cells. Actual values were 112 ± 4 and 49 ± 2 for RAW 264.7 cells and RAW.108 cells, respectively. Original magnification, ×20. **Significantly different (*p* < 0.01) from untreated cells.

Assays utilizing cells incubated with lysoPlsEtn showed that this simple treatment was enough to increase the capacity of RAW.108 cells to phagocytize the OpZ (Figure 4). The phagocytic capacity of normal RAW 264.7 cells was also augmented by lysoPlsEtn treatment, albeit the increase was lower, in relative terms, than that found for RAW.108 cells (Figure 4). Importantly, control experiments utilizing lysophosphatidylethanolamine (1-oleoyl-2-lyso-*sn*-glycero-3-phosphoethanolamine, lysoPtdEtn) instead of lysoPlsEtn, showed no effect on zymosan phagocytosis in either RAW 264.7 or RAW.108 cells (Figure 4), indicating specificity for the plasmalogen subclass; thus the presence of an alkenyl substituent at the *sn-1* position of the glycerol moiety appears to be necessary for the biological effect to manifest.

**Figure 5:**
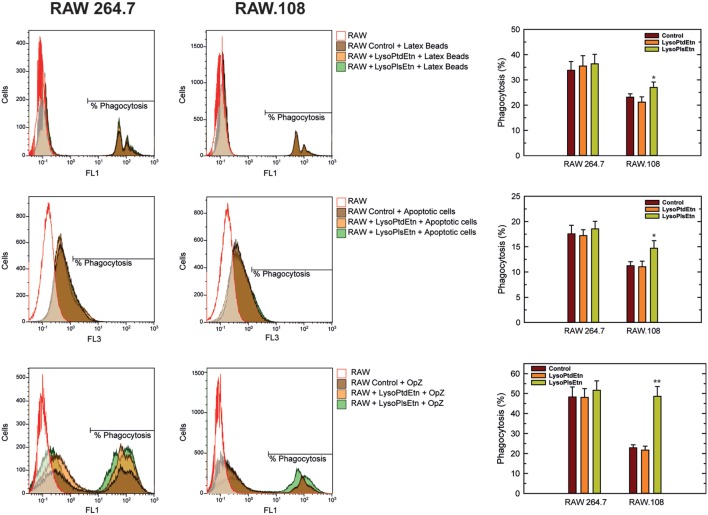
Effect of lysoPlsEtn on the phagocytosis of different targets by RAW 264.7 and plasmalogen-deficient RAW.108 cells. The cells were either untreated or treated with 10 µM lysoPlsEtn or 10 µM lysoPtdEtn for 10 min as indicated. Afterward, unopsonized latex beads (5–7 particles/cell, 30-min incubation) or apoptotic Jurkat cells (ratio 3:1, 2-h incubation) were added, and phagocytosis was analyzed by flow cytometry. Measurements run in parallel using opsonized zymosan particles (OpZ, 5–7 particles/cell) are also shown for direct comparison. *Significantly different (*p* < 0.05) from untreated cells; **significantly different (*p* < 0.01) from untreated cells.

To extend these results to other forms of phagocytosis, we assayed the effect of lysoPlsEtn on other phagocytic targets such as fluorescent latex beads (non-opsonized) and apoptotic cells. As shown in Figure 5, incubation of macrophages with lysoPlsEtn did significantly increase the capacity of RAW.108 cells to phagocytize both unopsonized latex beads and apoptotic cells; however, the extent of the effect was smaller than that found when OpZ was used as a target. We note that phagocytosis of apoptotic cells was not verified by confocal microscopy, so we cannot rule out that an undetermined amount of fluorescence arising from ingested material is due to phagocytized apoptotic cell debris. Collectively, these results suggest that while ethanolamine plasmalogens play a general role in regulating phagocytosis, their effect is particularly marked for opsonic phagocytosis.

**Figure 6:**
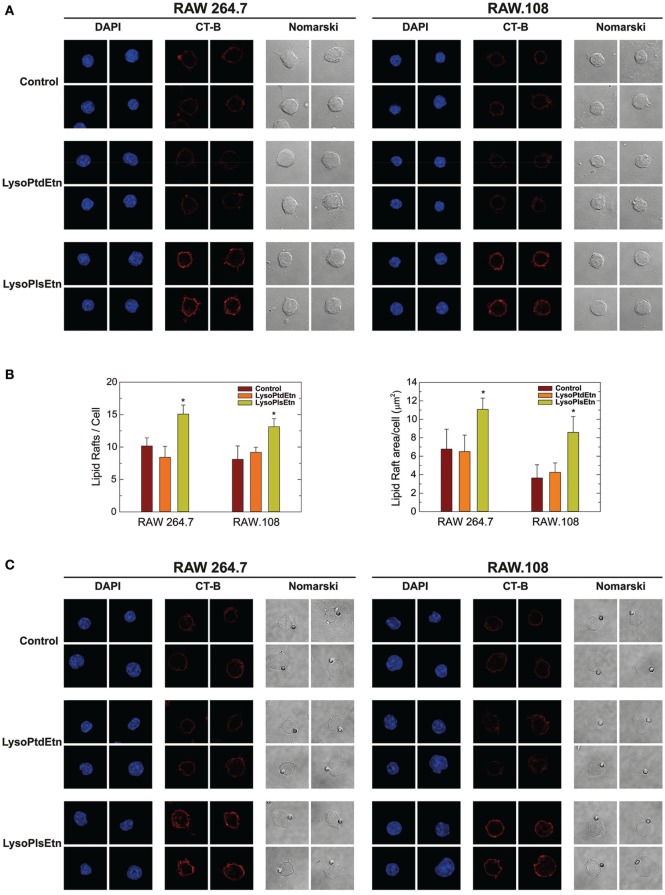

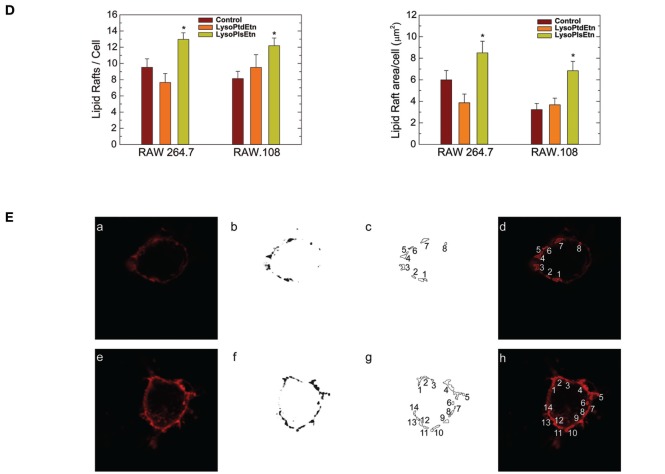
Effect of lysoPlsEtn on membrane rafts in RAW264.7 and plasmalogen-deficient RAW.108 cells. The cells were either untreated (brown bars) or treated with 10 µM lysoPlsEtn (orange bars) or 10 µM lysoPtdEtn (yellow bars) for 10 min. Then the cells were incubated for 30 min in the absence **(A,B)** or presence **(C,D)** of opsonized latex beads (10 particles per cell), stained with fluorescent cholera toxin (CT-B), and analyzed for lipid raft content by confocal microscopy (red color, middle columns). DAPI (1 µg/ml) was used to mark the nuclei (blue; left columns). Nomarski images are also shown (right columns). The average of three independent experiments with 20 determinations each is shown (mean ± SEM) (bottom panel). Original magnification, ×63. *Significantly different (*p* < 0.05) from untreated cells. **(E)** Methodology utilized to quantify lipid rafts: (a,e): native images marked with CT-B [(a) control RAW 264.7; (e) RAW 264.7 + lysoPlsEtn]; (b,f) threshold and transform into an 8-bit binary images with MaxEntropy algorithm of ImageJ Threshold tools software; (c,d,g,h) determination and quantification of lipid raft microdomains being equal or greater than 0.1 µm^2^ [(d,h) lipid raft overlay on native images].

Ethanolamine plasmalogens are frequent constituents of membrane lipid rafts (22). Thus, in the next series of experiments, confocal microscopy analyses of lipid rafts were carried out to determine the influence of plasmalogen content on these structures, as well as their relation with phagocytosis. Lipid rafts were visualized with Alexa Fluor 647-labeled cholera toxin B subunit (CT-B), which binds to ganglioside GM1 present in lipid rafts (17, 18). For the phagocytosis experiments, opsonized latex beads were used as stimuli instead of yeast-derived zymosan, because the autofluorescence of the latter interfered with the signal from Alexa Fluor 647-labeled CT-B. The results were similar in both resting (Figures 6A,B) and phagocytizing cells (Figures 6C,D); treating either RAW 264.7 cells or RAW.108 cells with lysoPlsEtn, but not lysoPtdEtn, increased both the number and size of lipid rafts in the two types of cells similarly. The methodology used to quantify the lipid rafts is shown in (Figure 6E). Collectively, these results suggest that enriching the cells with ethanolamine plasmalogen selectively increases both the number and size of lipid rafts on the plasma membrane, and the overall phagocytic capacity of the cells.

**Figure 7:**
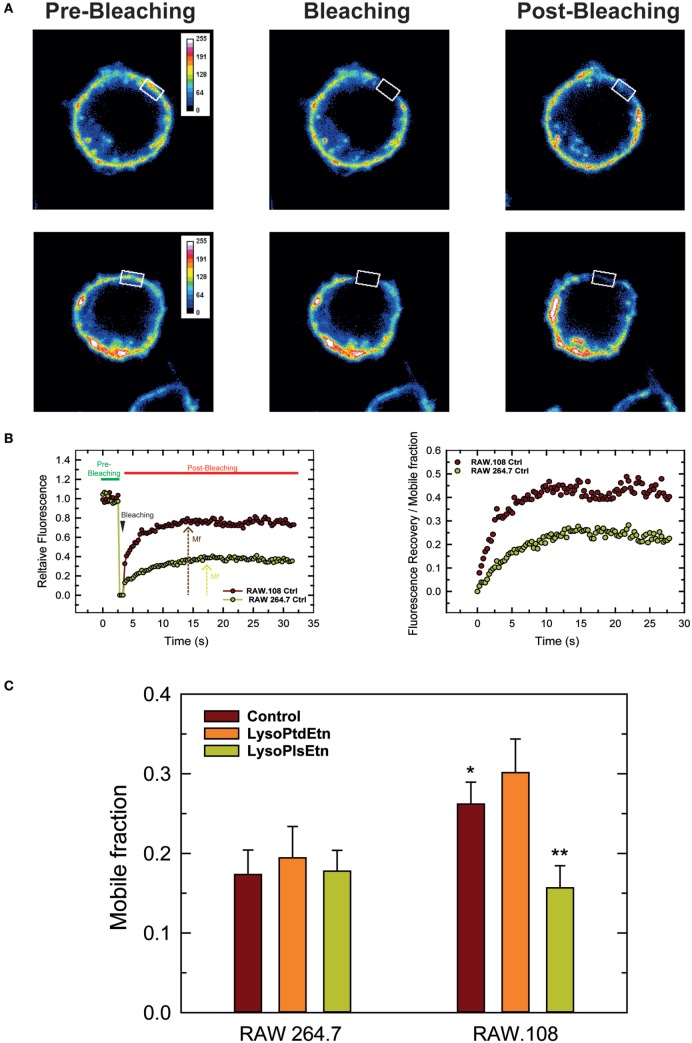
Analysis of membrane fluidity in RAW 264.7 and plasmalogen-deficient RAW.108 cells. The cells were either untreated (brown bars) or treated with 10 µM lysoPlsEtn (orange bars) or 10 µM lysoPtdEtn (yellow bars) for 10 min, were stained with fluorescent cholera toxin (CT-B) for fluorescence recovery after photobleaching (FRAP) *in vivo* by confocal microscopy. **(A)** During FRAP image acquisition, a number of images were collected before, during and after photobleaching of a defined region of interest (white square). **(B)** Left panel, quantification of the mean fluorescence in the regions of interest; an absolute intensity trace showing a typical bleach of 90–100% is illustrated. Right panel, normalized recovery after photobleaching, showing recovery of membrane fluorescence. **(C)** Comparison of the mobile fraction of membrane fluorescence after bleaching in RAW 264.7 and RAW.108 cells. Data in **(C)** are the average of three independent experiments of 40 determinations (mean ± SEM). **Significantly different (*p* < 0.01) with respect to untreated RAW.108 cells. *Significantly different (*p* < 0.05) with respect to untreated RAW264.7 cells. Original magnification, ×63.

Due to their chemical structure, plasmalogens are known to profoundly affect membrane fluidity (68, 69). To define whether cellular plasmalogen content is a key regulator of membrane biophysical properties, we carried experiments to analyze the membrane fluidity of RAW 264.7 and RAW.108 cells by FRAP using confocal laser microscopy (70, 71). Figure 7 shows that the membrane mobile fraction of RAW264.7 cells remains basically unaltered after treating the cells with lysoPlsEtn, which was to be expected since, as indicated previously, treating these cells with exogenous lysoPlsEtn only slightly increases the amount of cellular ethanolamine plasmalogen of the cells (Figure 3). Addition of lysoPtdEtn also had no effect. Untreated plasmalogen-deficient RAW.108 cells showed a significantly greater baseline membrane mobile fraction than normal RAW 264.7 cells, indicating that the membrane of these cells is more fluid than that of normal RAW 264.7 cells. Interestingly, when the RAW.108 cells were exposed to lysoPlsEtn but not to lysoPtdEtn, the membrane mobile fraction decreased, reaching values similar to those of normal RAW 264.7 cells. These data demonstrate that increasing plasmalogen levels in RAW.108 cells reduces cell membrane fluidity down to levels found in cells exhibiting normal plasmalogen levels, thus providing a rationale to explain the reduced phagocytic capacity of RAW.108 cells compared to normal RAW 264.7 cells.

**Figure 8:**
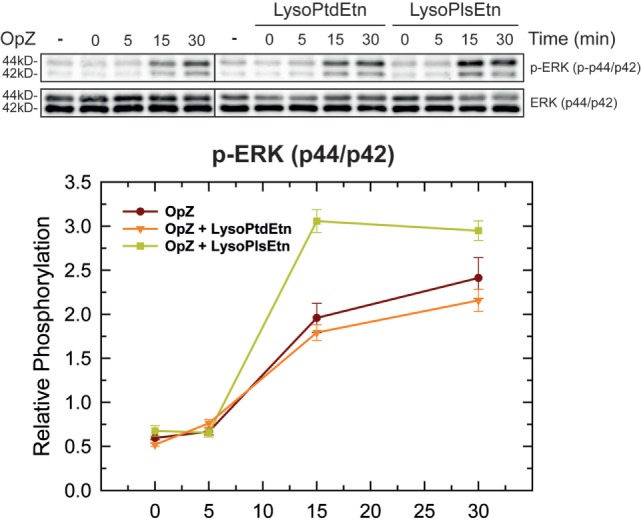
Effect of lysophospholipids on zymosan-induced signaling. RAW.108 cells were incubated with 10 µM lysoPtdEtn, 10 µM lysoPlsEtn or neither, as indicated, for 10 min. Afterward, the cells were stimulated with opsonized zymosan (OpZ) for the indicated times, and the phosphorylation of extracellular-regulated kinases (ERK) p44 and p42 was studied by immunoblot. The panel below the blots shows the relative quantification of p44 and p42 phosphorylation with respect to total ERK.

In order to clarify further how ethanolamine plasmalogens are involved in phagocytosis, we investigated whether lysoplasmalogen treatment of RAW.108 cells results in plasma membrane enrichment with phagocytic receptors. To this purpose, we analyzed the surface expression of CD11b, CD16, CD32, and CD64 by confocal microscopy using fluorochrome-coupled antibodies. No significant change in the expression of either of these receptors at the membrane, within or without lipid rafts, could be detected after treating the cells with lysoPlsEtn and OpZ. Thus these results suggest that surface expression of phagocytic receptors is not dependent upon plasmalogen content at the membrane. Importantly, however, we observed that lysoPlsEtn treatment of the RAW.108 cells significantly increased the phosphorylation activation of the extracellular-regulated kinases p44 and p42 by OpZ (Figure 8). These data suggest that plasmalogens bolster up the intracellular signaling that originates from phagocytic receptors, which may be instrumental to support an optimal phagocytosis response. Of note, treating the cells with lysoPtdEtn did not increase the zymosan-stimulated phosphorylation of p44/p42, highlighting the specificity of the lysoPlsEtn effect.

## Discussion

In this work, we show that plasmalogen deficiency in macrophages is associated with reduced phagocytosis. Such a reduction is significantly reversed when the cells are exposed to lysoPlsEtn, a strategy that restores in part cellular plasmalogen levels, especially the fraction containing arachidonic acid. The increase in the phagocytic capacity of OpZ experienced by RAW.108 cells after cellular plasmalogen restoration is accompanied by an increase in the number and size of lipid raft microdomains. While plasmalogen restoration may occur into the lipid draft, the possibility also exists that it takes place at other locations of the membrane, and still play a key role in the phagocytosis process. On the other hand, our results also raise the intriguing possibility that direct contact could exist between membrane plasmalogen and the opsonized target, this favoring phagocytosis of opsonized targets without the critical involvement of a phagocytic receptor. In this regard, recent work by Nakayama et al. (72) has demonstrated phagocytosis of mycobacteria by neutrophils *via* direct binding of bacterial lipoarabinomannan to lactosylceramide moieties in membrane lipid rafts.

Previous reports demonstrated that ethanolamine plasmalogens tend to accumulate in lipid raft microdomains (22), and are essential to maintain the stability of this structure (73). The unique biological properties of this class of phospholipids has been related to the presence of the *sn-2* vinyl ether link, which may influence membrane packing and, hence, some key biophysical properties such as fluidity, fusion tendency, and thickness (28, 29). It is thus relevant that in our study, exposing the cells to lysoPtdEtn has no effect on macrophage phagocytosis of OpZ, pointing out to the specificity of the plasmalogen vinyl ether moiety. The vinyl ether linkage at the *sn-1* position of plasmalogens allows the proximal regions of the *sn-1* and *sn-2* chains to become parallel, diminishing the distances between the carbons of these chains (68, 69). This enhances condensation and ordering of phospholipids in the membrane, resulting in decreased membrane fluidity (68, 69). As a result of this, ethanolamine plasmalogens form more condensed and thicker membranes than diacyl ethanolamine phospholipids, because the vinyl ether bond increased the thickness and decreased area per lipid by higher ordering of the *sn*-1 alkenyl chain, increasing the membrane packing and density (73). In perfect agreement with these data our FRAP analyses by confocal microscopy clearly indicate that increased plasmalogen levels in the cells decreases the fluidity of plasma membranes, i.e., increases membrane packing, and makes it comparable to that found in cells exhibiting normal plasmalogen levels. Rigidization of the plasma membrane by increased plasmalogen content may allow the formation of lipid raft microdomains, improve recruitment, oligomerization, and interaction of receptors and signaling proteins involved in phagocytosis.

Also related with plasmalogen structure, it is noteworthy that most of the lipid formed from exogenous lysoPlsEtn in our system contained arachidonic acid. While arachidonate-containing plasmalogen is indeed the major species found in normal RAW 264.7 cells, and also in primary macrophages (43), the former cells also contain measurable amounts of ethanolamine plasmalogen carrying oleic acid, dihomo-γ-linolenic, acid and several omega-3 fatty acids, all of which were formed only modestly in the lysoPlsEtn-treated cells. We speculate that the marked arachidonate preference for acylation of lysoPlsEtn in these cells is a direct consequence of the exceedingly high coenzyme A-independent transacylase activity (CoA-IT) that macrophage cell lines exhibit (26, 41, 60, 74–76). This enzyme catalyzes the selective incorporation of arachidonate moieties into ether lipids in a process directed to preserve membrane homeostasis by ensuring the appropriate distribution of this fatty acid among the various phospholipid pools (26, 77). The rapid action of CoA-IT likely depletes the lysoPlsEtn that has been provided to the cells, thus leaving no lysolipid available for acylation with other fatty acids *via* slower CoA-dependent reactions. From a biological point of view, this disparity in the formation of newly synthesized plasmalogen by the RAW.108 cells may explain why the recovery of the phagocytosis response is still lower than that observed in normal RAW 264.7 cells. Nevertheless, the recovery of phagocytosis, albeit incomplete, parallels the increase in number and size of lipid rafts in the membranes of the cells, suggesting that both events are closely related. Although arachidonate, as a polyunsaturated fatty acid, tends to increase the fluidity of the membranes to which it incorporates (78), our results clearly suggest that the thickening effect of the *sn-1* vinyl ether predominates over the fluidizing effect of the polyunsaturate at the *sn-2* position, since the net effect is a loss of membrane fluidity. It is possible that the enrichment of arachidonate in newly formed plasmalogens provides a counteracting force for finer regulation of changes of the biophysical properties of the membrane.

Due to their tendency to form non-bilayer structures, ethanolamine plasmalogens have also been found to facilitate membrane fusion and fission processes. These phospholipids reduce the transition temperature from non-lamellar to inverted hexagonal phase, exhibiting a high propensity to form an inverse hexagonal phase, which is essential for membrane fusion. Plasmalogens also reduce surface tension, which facilitates membrane fusion as well (79). In this regard, studies with fibroblasts derived from ether lipid-deficient human patients have reported marked decreases in exocytosis and endocytosis processes (80, 81). It has been found that, during phagocytosis, formation of the phagosome requires sealing of the phagosomal membrane *via* fusion processes that are in turn facilitated if the membrane is enriched with fusogenic lipids (82). Our results showing that restoring plasmalogen levels to RAW.108 cells increases phagocytosis are fully in accord with this view.

To conclude, our study provides novel data on the importance of plasmalogens in macrophage function and highlights the importance of ethanolamine plasmalogens in maintaining membrane fluidity and formation and organization of lipid rafts, and underscores their relevance for optimal cellular signaling that allows for an effective phagocytosis process. It is well established that the immune system deteriorates with age, which increases the risk of infections, autoimmunity, metabolic disorders, neurodegenerative and cardiovascular diseases, and cancer. Aging impacts on many macrophage processes such as phagocytosis, which may be related with the reduction of plasmalogen levels that is observed in these cells with aging (28, 83). Therefore, exogenous administration of plasmalogens could be considered as a valid strategy to both improve macrophage function and reduce the lipid composition imbalances seen with aging, so that the burden of age-related diseases can be alleviated. Future studies should explore this intriguing possibility.

## Ethics Statement

This study was approved by the Ethics and Bioethics Committee of the Spanish National Research Council (CSIC) prior to its commencement. No animals or samples of human origin were utilized for the experiments described in this report.

## Author Contributions

JR conducted experiments, interpreted the data, and wrote the manuscript. AA and JC conducted experiments and interpreted the data. MB designed the experiments and interpreted the data. JB designed the experiments, interpreted the data, and wrote the manuscript. All authors reviewed and approved the manuscript.

## Conflict of Interest Statement

The authors declare that the research was conducted in the absence of any commercial or financial relationships that could be construed as a potential conflict of interest.

## Abbreviations

lysoPlsEtn, lysoplasmenylethanolamine (1-*O*-1′-octadecyl-2-lyso-*sn*-glycero-3-phospho-ethanolamine); lysoPtdEtn, lysophosphatidylethanolamine (1-oleoyl-2-lyso-*sn*-glycero-3-phosphoethanolamine); FRAP, fluorescence recovery after photobleaching.
